# The Mediational Role of Coping Strategies in the Relationship Between Self-Esteem and Risk of Internet Addiction

**DOI:** 10.5964/ejop.v14i1.1449

**Published:** 2018-03-12

**Authors:** Rocco Servidio, Ambra Gentile, Stefano Boca

**Affiliations:** a Department of Languages and Education Sciences, Università della Calabria, Arcavacata di Rende, Italy; b Department of Psychological, Educational and Training Sciences, Università degli Studi di Palermo, Palermo, Italy; Department of Psychology, Webster University Geneva, Geneva, Switzerland; University of Wollongong, Wollongong, Australia

**Keywords:** self-esteem, coping, Internet addiction, university students, mediation model

## Abstract

The aim of the present study is to explore, through a mediation model, the relationship among self-esteem, coping strategies, and the risk of Internet addiction in a sample of 300 Italian university students. We submitted the data to a descriptive, mediational comparison between variables (t-test), and correlational statistical analyses. The results confirmed the effect of self-esteem on the risk of Internet addiction. However, we found that the introduction of coping strategies as a mediator gives rise to partial mediation. A low level of self-esteem is a predictor of avoidance-oriented coping that, in turn, affects the risk of Internet addiction.

The advent of the Internet and new media technologies changed different aspects of people’s way of life: for example, the organization of time (Mokhtari, Reichard, & Gardner, 2009), and the interaction with other people and with the social group of peers (Fioravanti, Dèttore, & Casale, 2012; Ruggieri, Boca, & Garro, 2013). Today, computer and mobile devices’ applications mediate the interaction among people, and communication technologies have become increasingly sophisticated and ubiquitous (Boca, Gentile, Ruggieri, & Sorce, 2013; Jones, 2002).

Although the Internet grants a number of advantages (e.g. faster information exchange, new communication forms), some habits, such as online gaming and gambling, or the use of social networking sites could be so persistent that it could turn into a real addiction to the Internet.

Internet Addiction Disorder (IAD) is generally described as an individual’s incapacity to control and manage the time and manner of using the Internet. Such incapacity may give rise to behavioural disorders (Young, 2017). Currently, there are no standard diagnostic criteria for accurately identifying individuals with IAD. However, for the purpose of the present study we agree with Griffiths (1995) who defines IAD as an addictive behaviour characterized by behavioural dominance, mood alteration, tolerance, abstinence and conflicts.

In an attempt to cover this diagnostic gap, different proposals have been advanced (Beard & Wolf, 2001; Griffiths et al., 2016; Panayides & Walker, 2012). Young and Abreu (2011), for example, maintain that a method for clinically detecting the compulsive use of Internet consists in the application of existing criteria on addiction behaviours as cited in the last edition of the Diagnostic and Statistical Manual of Mental Disorders- Fifth Edition (DSM-V; American Psychiatric Association, 2013).

Recent studies highlight that individuals who are not able to control the amount of time spent on the Internet also tend to exhibit difficulty in stopping compulsive behaviours (Zhang et al., 2015). They also exhibit personality traits that can increase the individual’s vulnerability (Servidio, 2014; Yao, He, Ko, & Pang, 2014) and other behavioural disorders (Kuss & Lopez-Fernandez, 2016).

The use of Internet, however, is not the principal cause of the addiction but is an instrument through which people try to achieve a state of wellness (Griffiths, Kuss, Billieux, & Pontes, 2016).

Recently, researchers have focused their attention on the new generation of Internet users, among whom are university students, who may be considered at risk for developing pathological behaviours since they are involved daily in the use of different kind of technologies (Bahrainian, Alizadeh, Raeisoon, Gorji, & Khazaee, 2014; Coniglio, Sidoti, Pignato, Giammanco, & Marranzano, 2012; Servidio, 2014).

Among the factors known to contribute to the onset of the IAD, the most relevant ones concern personality traits (Kuss & Lopez-Fernandez, 2016), as well as the series of one’s major life events (Li et al., 2016; Zhou, Li, Li, Wang, & Zhao, 2017). Besides personal history, stable features of the self are known to be related to Internet addiction. For instance, a number of studies reveal that people with low levels of self-esteem tend to access the Internet largely than those with higher self-esteem levels (i.e. Andreassen, Pallesen, & Griffiths, 2017; Bozoglan, Demirer, & Sahin, 2013). Thus, self-esteem, insofar as it influences the compulsive use of new technological instruments, may be considered a good predictor of the risk of the IAD.

Self-esteem is defined as the positive or negative attitude toward the Self (Coopersmith, 1967). People with high levels of self-esteem tend to use technology in a balanced manner, tend to cope positively with stressful situations, and develop a positive attitude toward life (Taylor & Brown, 1988). On the contrary, people with low levels of self-esteem try to find shelter in the Internet network, which allows them to control the Self-aspects they want to make public. Similar to what is commonly observed with other forms of addictive behaviour, low levels of self-esteem should be associated with the IAD. Andreassen, Pallesen, and Griffiths (2017) directly tested this hypothesis, and found that participants’ self-esteem is negatively correlated with the compulsive use of social media applications.

An increase in Internet usage have been also documented in college students who had gone through adverse life events (e.g. academic failures, romantic break-up, difficulties with parents, etc.) compared to students that live under the family’s constant supervision throughout the day (Tian, Bian, Han, Gao, & Wang, 2017). For this reason, it is interesting to analyse the role of coping strategies since they allow people to master their own behaviour and successfully face stressful life events. Thus, differences in prevailing coping strategies should be associated to changes in the propensity for Internet addiction.

The term “coping” includes cognitive, emotional, and behavioural strategies that individuals use when they are in stressful situations in order to control associated unpleasant experiences (Folkman & Lazarus, 1988; Spagna, Russo, & Roccato, 2014). Those strategies are commonly classified into three categories: appraisal-focused, emotion-focused, and problem-focused (Endler & Parker, 1994).

Few researches have explored the role that the coping strategies may play in predicting the risk of Internet addiction (Deatherage, Servaty-Seib, & Aksoz 2014; McNicol & Thorsteinsson, 2017). The results of these studies point out that IAD is systematically linked to avoidance coping, a way of coping that is characterized by the effort to avoid dealing with a stressor.

Cyberspace may represent a place to hide when things go wrong. In this sense, accessing the Internet can be considered a negative coping strategy in facing stressful events. An excessive use of such a strategy may result in new forms of technology addiction because individuals, in the desire to escape from reality, completely avoid the source of stress (Tang et al., 2014). The results of a recent integrative review show that personal dispositional coping style predicts problematic computer use in adolescence (Trnka, Martínková, & Tavel, 2016). The authors of the review underlined the importance of further investigations because the current results indicate a higher differentiation of coping strategies in Internet addiction, and this relationship is still not sufficiently covered by present studies.

Most of the present Italian studies, in addition, focus on the predictors of the risk of Internet addiction, thus leaving unexplored the variables that might mediate this relationship. The present study aims to fill this gap by testing a model for analysing the mediating effects that coping strategies have on the relationship between self-esteem and Internet addiction. We propose an integrated model of the risk of Internet addiction, providing some empirical evidences about the complex nature of this disorder.

## Aims and Hypotheses

The present study aims at investigating, through a mediation model, the role of coping strategies in the relationship between self-esteem and Internet addiction disorder. Previous studies have confirmed that people with low levels of self-esteem spend more time on the Internet, and are more susceptible to be addicted to information and communication technologies (Bozoglan et al., 2013; Yao et al., 2014).

Another frequently discussed issue concerns the role of the coping strategies. Individuals who have difficulty in coping with stressful real-life events are more likely to develop Internet addiction compared to others who can cope better with these stressful incidents (Chou et al., 2015).

Generally speaking, self-esteem and coping strategies seem to be systematically related: individuals with low self-esteem tend to adopt avoidance strategies when facing stressful events (Chapman & Mullis, 1999). To date, there are scarce evidences in literature that coping strategies mediate the link between self-esteem and Internet addiction. A study by Chiu (2014) showed that self-efficacy was a mediator between academic stress and smartphone addiction; another study has pointed out that poor coping and cognitive expectations mediate the relationship between generalized Internet addiction and factors such as self-efficacy and stress (Brand, Laier, & Young, 2014).

The above-discussed results allow the definition of the following research hypotheses: self-esteem is an indirect predictor of the risk of Internet addiction; low levels of self-esteem will increase the use of avoidance strategies in the students, and these strategies in turn will increase the vulnerability to dysfunctional behaviour when using Internet in a maladaptive way.

## Method

### Participants

A sample of 300 university students, who lived in the residential area of the campus, took part in this research. Participants were 125 males aged 18-30 years (*M* = 23.82, *SD* = 3.42), and 175 females aged 18-30 years (*M* = 23.61, *SD* = 3.16) from humanistic (26.3%), scientific (22.3%), and economic courses (18.0%). Informed consent was obtained for all participants before the beginning of the assessment.

Participants declared that they connect to the Internet for at least 3 hours per day (36.7%). They generally use their smartphone to connect to the Internet (57.7%) followed by notebook/PC (41.0%). The preferred time for connecting to the Internet is the evening (38.0%) and the afternoon (32.7%). Concerning communication, people use the Internet for controlling their own Facebook account (53.3%), for chatting (21.3%) and emailing (19.3%). Finally, participants declared not to perceive their own behaviour as addictive (79%).

### Measures

#### Demographic Information

A subject’s profile was designed to include questions such as gender, educational background, and other relevant information. Then data about their Internet activities based on time spent online per day, type of online activities engaged daily (communication, surfing, and interactive activities), and the preferred time to establish a new Internet connection (morning, afternoon, evening and night) were obtained through a multiple-choice modality.

The t-test results showed that males spend more time on the Internet compared to females (*M*_male_ = 1.98, *SD* = 1.13, *M*_female_ = 1.71, *SD* = 1.15; *t*(298) = 1.97, *p* < .05, *d* = .23). In addition, the risk of being addicted to the Internet is higher in the male students than in females (*M*_male_ = 43.29, *SD* = 12.24, *M*_female_ = 39.51, *SD* = 11.04; *t*(298) = 2.78, *p* < .05, *d* = .32).

#### Self-Esteem

The Italian version of the Rosenberg Self-Esteem Scale (Prezza, Trombaccia, & Armento, 1997) was used to measure the subjective feelings of self-value and self-acceptance. Participants rated their agreement with 10 statements (e.g. “I feel that I have a number of good qualities”) on a scale of 1 (strongly disagree) to 4 (strongly agree). Higher scores indicate higher self-esteem. The internal consistency in the sample of this present study was good (α = 0.83).

#### Coping Orientation to Problems Experienced - New Italian Version (COPE-NIV)

The COPE-NVI (Sica et al., 2008) is a 60-item measure of how people respond when they deal with difficult or stressful events in their lives. Every item is rated on a 4-point Likert scale ranging from 1 “I usually don't do this at all “and 4 “I usually do this a lot”. Its five subscales include social support (e.g. I try and get advice from someone about what to do), avoidance strategies (e.g. “I say to myself this isn’t real”), positive attitude (e.g. I restrain myself from doing anything too quickly), problem solving (e.g. I concentrate my efforts on doing something about it), and turning to religion (e.g. I seek God’s help).

Higher scores in a subscale indicate that participants tend to cope with stress by using those strategies. The internal reliability of the COPE-NVI in the present study was good (α = .83), whereas the alpha’s value for each subscale was for social support (α = .88) avoidance strategy (α = .84), positive attitude (α = .63), problem solving (α = .73) and turning to religion (α = .75).

#### Young Internet Addiction Test (IAT)

We used the self-administrated Italian version of the Internet Addiction Test (IAT) to assess the participants’ severity of Internet addiction risk (Servidio, 2017). The IAT includes 20 items rated on a five-point Likert scale (i.e. “How often does your job performance or productivity suffer because of the Internet?”). The response format ranges from 1 (Rarely) to 5 (Always), and the overall score can range from 20 to 100 (cut-off is 80), with higher scores reflecting greater life and social problems caused by a misuse of Internet services. For the current study, the Cronbach’s alpha measure demonstrated good reliability (α = .90).

### Procedure

All the study participants were recruited individually throughout the university campus during the lesson breaks. The research assistant explained to the participants the general aim of the study, as well as the procedures and the methods of completing all the measures. Participants were also informed that their participation was voluntary, anonymous, and without any incentive. In order to encourage honest reporting, the anonymity of the study was emphasized at the beginning of each collection session. After receiving the informed consent from the participants, the research assistant administered the questionnaires to collect the data, which took approximately 20 minutes. All the research materials and study procedures were designed according to the Ethics in Human Research (Associazione Italiana di Psicologia, 2015).

Finally, the collected data were analysed via the software SPSS v.23.0. The multiple mediation effect in this study was tested using the macro PROCESS for SPSS through model 4 (Hayes, 2013). The bootstrapping method was used to test for the significance of the effect in order to obtain robust standard errors for parameter estimation. It produced 95% bias-corrected confidence intervals of these effects 10000 resamples of the data. In addition, t-test for independent samples was ran to verify differences among the main variables of the current study and correlation analyses were computed.

## Results

### Demographic Characteristics

By applying Young’s (1998) IAT cut-off criteria based on the severity impairment risk-index, the results showed that 60 students (20.2%) used Internet correctly (scores 0-30). On the other hand, 179 students (59.3%) surfed the web longer than was usually called for, although they retained control over their online sessions (scores 31-49). The remaining 62 (20.7%) of the participants showed a moderate risk of experiencing occasional or frequent problems due to Internet usage, which should warn these subjects about the Internet’s negative impact on their life (scores 50-79). For this current study, no severe Internet addiction risk (scores 80-100) was found among the participants.

### Mediation of Coping Strategies

Table 1 displays the correlations between the study variables. All the variables were significantly correlated in the expected directions.

**Table 1:** Inter-Correlations and Descriptive Results

Variable	*M*	*SD*	1	2	3	4	5	6	7
1. Self-Esteem	30.23	5.14	–						
2. Internet Addiction	41.08	11.72	-.25**	–					
3. Social Support	29.50	7.45	-.12*	.11	–				
4. Avoidance Strategies	26.80	7.35	-.36**	.32**	.17**	–			
5. Positive Attitude	30.25	4.68	.00	.05	.14*	.10	–		
6. Problem Solving	31.45	5.31	.14*	.03	.23**	-.10	.32**	–	
7. Turning to Religion	18.92	4.61	-.05	.03	.13*	-.09	.01	.01	–

*Note*. Sample = 300.

*p < .05. **p < .001.

**Figure 1:**
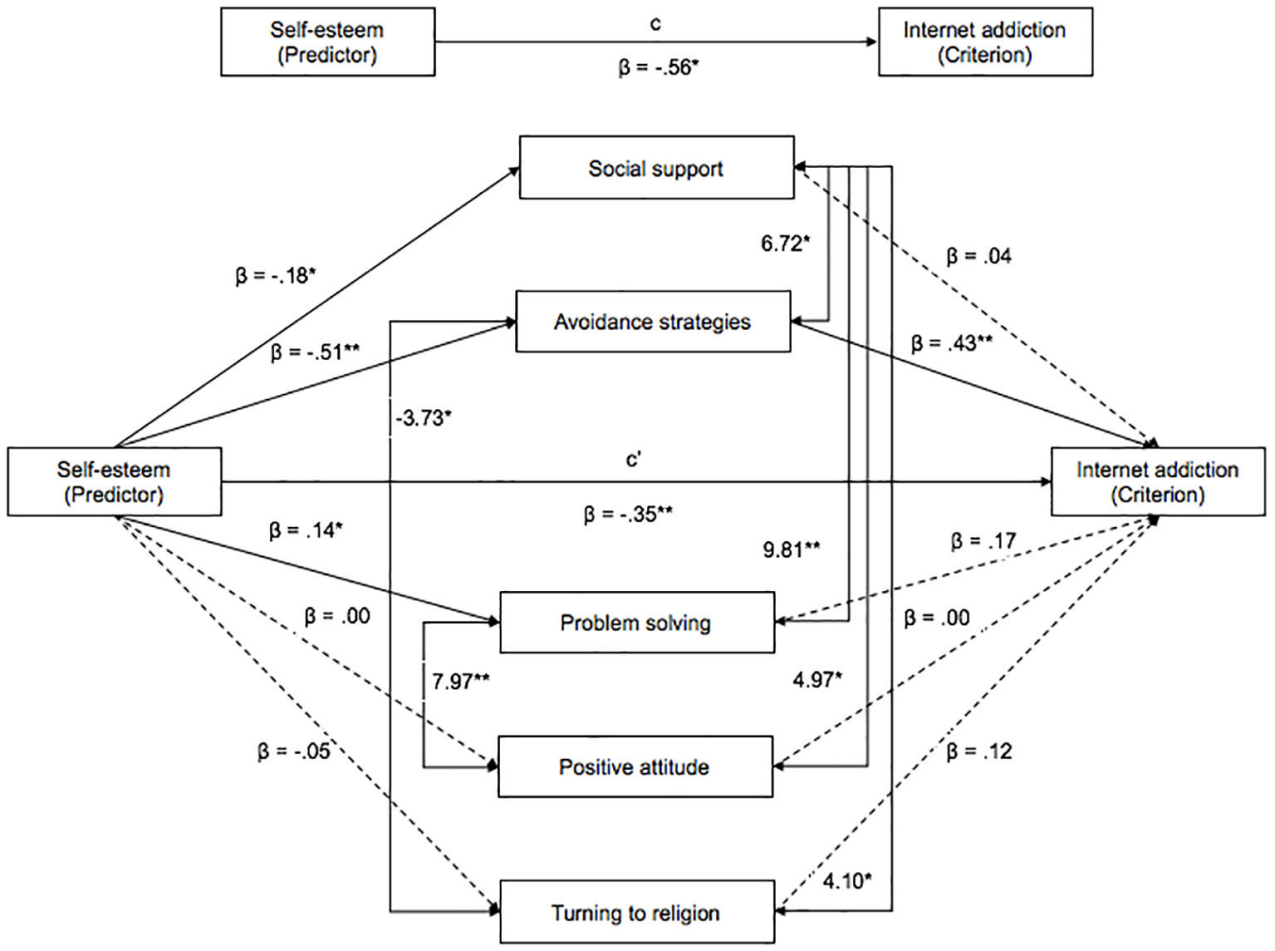
Visual representation of the mediation model including unstandardized results. *Note*. The model reports both the significant (continuous lines) and not significant (dotted lines) relationships. The covariance relationships among the coping variables and the related values are depicted as well. *p <.05. **p < .001.

After this preliminary assessment, we evaluated the partialisation of the common variance through a direct test of the hypotheses by applying a mediation model. The results of the mediation analysis are showed in Figure 1.

First of all, a significant total effect of self-esteem on Internet addiction was found [c] (*t* = -4.53, *p* < .001; *B* = -.56, *SE* = .12, 95% CI [-.77, -.24]). Among all the coping strategies, only avoidance strategies mediated the relation between self-esteem and Internet addiction. In fact, avoidance coping was predicted by self-esteem (*t* = -5.64, *p* < .001; *B* = -.51, *SE* = .09, 95% CI [-.67, -.29]) and the Internet addiction risk, in turn, was predicted by avoidance strategies (*t* = 4.34, *p* < .001; *B* = .43, *SE* = .10, 95% CI [.26, .68]). This result means that people with low levels of self-esteem tend to spend time on the Internet as a strategy to cope with stressful events. Concerning the other coping strategies, although social support and problem solving are significantly predicted by self-esteem, they do not predict, in turn, the risk of Internet addiction.

The direct effect [c’] shows that avoidance strategies remain significant after partialising out the variance explained by coping strategies, thereby obtaining a partial mediation result (*t* = -2.60, *p* < .05; *B* = -.35, *SE* = .13, 95% CI [-.62, -.09]).

Furthermore, a significant indirect effect of self-esteem on Internet addiction through avoidance strategies was found (*t* = -3.76, *p* < .001, *B* = .22, *SE* = .06, 95% CI [-.33, -.09]). A Sobel test was conducted to confirm the partial mediation in the model (*z* = - 3.69, *p* < .05).

Finally, the 39% of the total indirect effects of self-esteem on Internet addiction is mediated by avoidance coping.

## Discussion and Conclusion

The current study examines the mediation effect of coping strategies in the relationship between self-esteem and the risk of Internet addiction. As hypothesized, the results obtained through the mediation analysis show that the effect of self-esteem is influenced by coping style, in particular by avoidance coping, that is, the degree to which people engage in actions such as negation, humour, substance abuse, behavioural and mental disengagement. Low levels of self-esteem, in fact, are associated with a major use of avoidance coping that is pathologically expressed with the abuse of the Internet. However, the results suggest a partial mediation effect. Thus, people with low self-esteem are more at risk of developing a kind of “new-media psychopathology”, where the Internet is the main tool of communication.

On one hand, the current study puts the results in line with literature, confirming self-esteem as predictor of the risk of Internet addiction (Andreassen et al., 2017; Bozoglan et al., 2013; Zhang et al., 2015). On the other hand, it explains the way by which self-esteem effects are mediated by the tendency to make use of coping strategies. Not all the coping strategies are involved in this relation: in fact, only the strategies based on avoidance play a significant role. Social support, for example, did not reach any significance, in contrast with the results of Naseri, Mohamadi, Sayehmiri, and Azizpoor (2015).

Therefore, it is clear that people who are more at risk for Internet addiction seem to be those who use the Internet as a regular daily object of gratification for personal needs. Nevertheless, it should be noted that the result of the current study provides only a partial view of the link between self-esteem and the Internet addiction since coping strategies represent only one of the different factors that are able to mediate this relationship.

Another limitation of the present study concerns the specificity of the sample selected. University students are commonly a well-adapted population, therefore no clinical sample affected by pathological addiction to the Internet took part in this study. Thus, there is no evidence about possible direct or mediational effects of self-esteem and coping strategies for those who are seriously unable to control their Internet behaviour. To adequately generalize what was obtained here, these results should be replicated with a larger and stratified sample. Despite these limitations, however, the present study is the first to investigate the relation between self-esteem and the Internet addiction, linking this relation with participants’ coping style. If high levels of self-esteem, associated with a low appeal for avoidance strategies, are factors that protect from the risk of Internet addiction, these results can be used as guidelines in academic counselling. Furthermore, understanding what are the risks factors of Internet addiction could help in preventing the risk of some psychological consequences, such as avoidance of responsibilities, personality changes, social alienation and the risk of other forms of addictive behaviours (Young, 2004).

Future research might investigate other potential predictors and mediators in the relation between self-esteem and Internet addiction, such as perceived stigma, resilience, and affective relations. In this way, new evidence-based strategies could be defined for the promotion of wellness and the prevention of Internet misuse.
